# Control of the mixing time in vessels agitated by submerged recirculating jets

**DOI:** 10.1098/rsos.171037

**Published:** 2018-01-24

**Authors:** Stephen Kennedy, Pradipto K. Bhattacharjee, Sati N. Bhattacharya, Nicky Eshtiaghi, Rajarathinam Parthasarathy

**Affiliations:** 1 Chemical and Environmental Engineering, School of Engineering, RMIT University, Melbourne, Victoria 3001, Australia; 2 Rheology Solutions Pty Ltd, 15-19 Hillside St., Bacchus Marsh, Victoria 3340, Australia

**Keywords:** jet mixing, ERT, suction, non-Newtonian mixing

## Abstract

Submerged recirculating jet mixing systems are an efficient and economical method of agitating large tanks with a high hydraulic residence time. Much work has been carried out in developing design correlations to aid the predictions of the mixing time in such systems, with the first such correlation being developed nearly 70 years ago. In most of these correlations, the mixing time depends directly on the volume of the vessel and inversely on the injection velocity of the submerged jet. This work demonstrates, for the first time, that the distance between the injection and suction nozzles also significantly affects the mixing time and can be used to control this time scale. The study introduces a non-dimensional quantity that can be used as an adjustable parameter in systems where such control is desired.

## 1. Introduction

Jet mixing is a single-phase mixing process, whereby a high velocity jet of fluid entrains the surrounding fluid, creating a mixing layer at the jet boundary. Jet mixing is commonly employed in large-scale tanks where the required blend time of the tanks is more in the order of hours rather than minutes or seconds. Therefore, jet mixing is ideal for anaerobic digesters, fuel storage tanks, etc. with hydraulic retention times in the order of days. As jet mixing does not require any moving parts inside the mixing tank, it is easy to maintain, and considered more economical than other forms of mixing . (The nomenclature used in this article is provided in table 1.)

**Table 1.** Nomenclature.

**Greek alphabet**	
$\dot{\gamma }$	shear rate (s^−1^)
Δ*S*	distance between the centre lines of the jet and suction nozzles (m)
*Λ*	non-dimensional design ratio
Λ_*_	ratio of distance between nozzles to diameter of suction nozzle
*η*_app_	non-Newtonian apparent viscosity (Pa s^−1^)
*μ*	Newtonian viscosity (Pa s^−1^)
*ρ*	fluid density (kg m^−3^)
*σ_i_*	Individual normalized conductivity measurements (*C_i_*_(*t*)_/*C*_ref_)
σ*	threshold normalized conductivity value
*τ*_y_	yield stress (Pa)

	Latin alphabet
*a*	Meyer & Etchells [13] modelling constant
*A*_1_, *A*_2_	fitting parameters for design equation [14]
*c*	concentration of NaCl in the vessel (g l^−1^)
*C_i_*_(*t*)_	individual conductivity measurements taken at time, *t* (mS cm^−1^)
*C*_ref_	reference conductivity taken at the beginning of ERT experiments (mS cm^−1^)
*d*_j_	diameter of jet nozzle (m)
*d*_s_	diameter of suction nozzle (m)
*D*_T_	tank diameter (m)
*f*	Fox & Gex [2] correlation factor
*Fr*_j_	jet Froude number
*g*	gravitational constant (m s^−2^)
*H*	height of the observed mixing chamber (m)
*H*_c_	height of cavern (m)
*H*_T_	liquid height in mixing tank (m)
*l*_s_	average length-scale of the suction domain of influence (m)
*k*	flow consistency index (Pa.s^n^)
*n*	flow behaviour index
P1–4	tomographic sensor planes from 1 to 4
$\dot{Q}$	recirculation flow rate (m^3^ s^−1^)
*r*_a_	time-dependent radius of the active region boundary (m)
*r*_T_	radius of tank (m)
*Re*_j_	jet Reynolds number
*Re*_j−PL_	power-law jet Reynolds number
*T*	process time (s)
*t_θ_*	mixing time (s)
*t*_H_	hydraulic residence time (s)
*V*	volume of observed mixing chamber (m^3^)
*V*_a_	active mixing volume (m^3^)
*V_i_*	inactive mixing volume (m^3^)
*V*_T_	tank volume (m^3^)
*v*_j_	jet velocity (m s^−1^)
*v*_s_	velocity of suction (m s^−1^)

Submerged jets in tanks can either occur radially, through a side entrance of the tank, or axially, along the axis of the tank. The study of submerged jet mixing in tanks has historically focused on mixing time, *t_θ_*, as the key criterion for measuring mixing success. One of the first in-depth studies of large-scale jet mixing of Newtonian fluids was by Fossett & Prosser [1], investigating the effect that free jets (jets unbounded by vessel geometry) have on the blend time of tetraethyl lead with aviation fuel in underground storage tanks. Their experiments consisted of a 1.5 m diameter tank (*D*_T_), with a liquid height (*H*_T_) of 0.9 m fitted with an inclined side-entry nozzle. They used a single nozzle with diameters between 0.02 and 5.7 mm (*d*_j_) with jet Reynolds numbers (*Re*_j_) between 4500 and 80 000, where

1.1 $$Re_{j}=\frac{d_{j}v_{j}\rho }{\mu },$$

where *v*_j_ is the nozzle injection velocity (m s^−1^), *ρ* is the fluid density (kg m^−3^) and *μ* is the Newtonian viscosity (Pa.s^−1^). *Re*_j_ < 100 signifies laminar flow, while *Re*_j_ > 1000–2000 signifies fully turbulent flow, placing Fossett and Prosser's research firmly in the turbulent regime. They proposed the following correlation for blend time:

1.2 $$t_{\theta }=9\frac{D_{T}^{2}}{v_{j}d_{j}}.$$

Fox & Gex [2] extensively investigated the effects of both side-entry jets and propellers in water, glycerol and cooking oil, using an acid–base tracer technique in 0.305, 1.52 and 4.27 m diameter tanks. They were able to correlate mixing time for all three scales that varied with flow regime within ±50% such that

1.3 $$t_{\theta }=f{\left(\frac{Fr_{j}}{Re_{j}}\right)}^{1/6}\frac{D_{T}H_{T}^{1/2}}{d_{j}^{3/2}},$$

where *Fr*_j_ is the jet Froude number, a dimensionless number equal to *v*_j_*^2^/g.d*_j_, where *g* is the gravitational constant, *f* is a correlation factor that is dependent on flow regime and a different factor is used depending on laminar or turbulent regimes.

Van De Vusse [3] tested both models in a 32 m diameter tank (*H*_T_ = 13 m) agitated by a side-entry jet with a nozzle diameter of 0.050 m at a 25° angle, and found that their data best fit Fossett and Prosser's [1] correlation such that

1.4 $$t_{\theta }=3.6\frac{D_{T}^{2}}{v_{j}d_{j}}.$$

Additionally, Okita & Oyama [4] contended that the scatter found in Fox and Gex's work was too great and set out to find their own correlation. Using inclined side-entry jets, they posited that the nozzle angle had no effect on mixing time, and mixing time in a fully turbulent tank (*Re*_j_ > 7000) is independent of Reynolds number. Their correlation was as follows:

1.5 $$t_{\theta }=5.5\frac{D_{T}^{3/2}H_{T}^{1/2}}{v_{j}d_{j}}.$$

Interestingly, Okita & Oyama [4] had placed the suction nozzle of their recirculation system just behind the inclined side-entry jet. However, the effects of the interaction between the jets on the flow patterns were not discussed. Remarkably, at the time when the such interaction was typically ignored, Fox and Gex alluded to the phenomenon in acknowledging that jet placement is not critical to mixing time provided that it does not feed directly in the suction nozzle. Nevertheless, the effect of jet placement on mixing time remained controversial.

A summary of the correlations for jet mixing performance can be found in Patwardhan & Gaikwad [5]. It can be observed from the discussion there that the majority of jet mixing studies to date have been in Newtonian fluids that ignore the effect of suction. This is most probably due to the viscosity of the Newtonian fluids used remaining constant throughout the bulk of the fluid, thus it is not likely to experience different rates of deformation depending on inlet and suction point. Revill [6] carried out another well-researched review, and gave design recommendations, mentioning that the injection nozzle should be placed as far away from the suction nozzle as possible. While this is perhaps intuitive, it is unclear how this conclusion is reached. In their extensive study of jet placement, Orfaniotis *et al*. [7] keep the location of the suction nozzle constant, while varying most other factors. A key assumption in this work followed from previous research which suggested that the position of the nozzle does not significantly influence the flow structure in the reactor which only depends on jet momentum. Jayanti [8] studied the interaction using CFD and found that in areas with strong recirculation currents jet mixing is advection-dominated, while in regions of low recirculation currents jet mixing is diffusion-dominated. It was suggested that increasing the strength of recirculation currents does not necessarily lead to improved mixing, and it was found that mixing is optimized when a balance between advection and diffusion is found.

Studies on non-Newtonian media in jet recirculating systems are not common in the literature although some examples do exist [9–12]. Studies on pulse jets in non-Newtonian media are more developed. Meyer & Etchells [13] expanded on Solomon *et al*.’s [14] concept of the cavern, that is, a steady-state condition in which an active mixing region that extends only to the point where local stresses imposed by the mixing source become equal to the yield stress of the fluid. Once the cavern has reached its equilibrium volume, it remains fixed. Yield stress is central to the concept of the cavern, and Meyer and Etchells derived the following relationship to describe cavern dimensions:

1.6 $$\frac{H_{c}}{D_{T}}=\frac{av_{j}}{d_{j}}\sqrt{\frac{\rho }{\tau_{y}}}-\frac{1}{2},$$

where *H*_c_ is the height of the cavern (m), *τ*_y_ is the yield stress (Pa) and *a* is a model constant that is a weak function of *Re*_j_. Pulse jets have the geometric simplicity of having one energy source, thus deriving a relationship describing the dimensions of a cavern is less challenging than recirculating jets where suction may play a role. The authors have shown previously that cavern formation can occur in yield stress fluids when agitated by submerged recirculating jets [15]. While using electrical resistance tomography to explore the three-dimensional nature of the tank, it was revealed that suction played a greater role than previously reported in the literature. The effect of suction resulted in an asymmetrical cavern structure, without a characteristic dimension described in Solomon *et al.* [14] or Meyer & Etchells [13]. To compensate for this, a new technique for measuring asymmetric volumes was developed.

The picture that emerges is that while it was known from the past research that the positioning of the nozzles might impact the mixing time in vessels agitated by recirculating liquid jets, the nature of the interactions is not clear. The situation is even more complex in cases where non-Newtonian media are used, as the coupling between the rheological response of the liquid and the fluid mechanics can be complicated and has not been studied in detail. The developments of caverns in agitated vessels that process liquids with a yield stress are also interesting, and it is unclear what role the placement of the nozzles plays in determining the volume of the caverns. These aspects remain open to further research and we address a few of them here. We are particularly interested in understanding the role that the suction nozzle plays in the mixing of the liquids and how the mixing time changes with nozzle placement in non-Newtonian liquids (without a yield stress) agitated with recirculating liquid jets.

Jet mixing in tanks with non-Newtonian media is industrially relevant in a number of fields, for example, anaerobic digesters in waste water treatment, where the rheology is shear thinning and may or may not contain a yield stress. In other industrial applications, different rheology must be considered, such as jet mixing in nuclear waste storage which must take into account the significant yield stress of the material, or mixing polymer solutions where the rheology can be even more complex.

## 2. Material and methods

All experimental runs were conducted in a 20 cm diameter tank with a 1 : 1 aspect ratio (*D*_T_ *= H*_T_ *= * 0.20 m). The working fluid used was 0.3 wt% xanthan gum Keltrol T solution (XGKT). The rheological characteristics of the XGKT solution were measured using a HR3 Discovery rheometer (TA Instruments, USA) with a cone-and-plate geometry using a 60 mm diameter 2° cone under ambient conditions. The fluid is drawn out of the tank through a 6 mm diameter (*d*_s_ = 0.006 m) suction nozzle using a peristaltic pump (Masterflex L/S, Cole-Parmer, USA) and recirculated back into the tank through a 6 mm diameter jet nozzle (*d*_j_ =  0.006 m). All runs in this study use a jet nozzle velocity of 0.81 m s^−1^ (*v*_j_ = 0.81 m s^−1^). The main design variable manipulated in this study includes the placements of the jet and suction nozzles.

**Figure 1.**
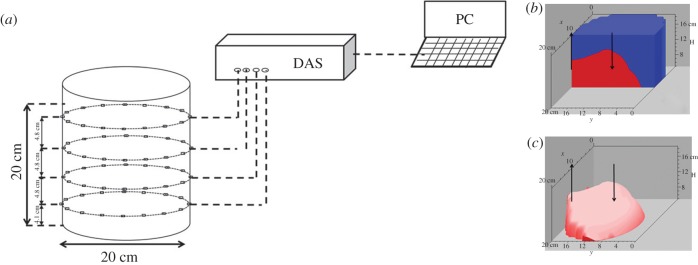
(*a*) Schematic of ERT system used to monitor mixing quality. (*b*) A representative 3D Slicer-Dicer reconstruction of the mixing environment in the tank. (*c*) A representative 3D Slicer-Dicer reconstruction of the mixing environment in the tank with the well-mixed active region isolated.

The method of measuring mixing performance was first explained in Kennedy *et al*. [15]. In summary, mixing performance was measured using a saline tracer and an electrical resistance tomography (ERT) system supplied by Industrial Tomography Systems (ITS, Manchester, UK), a schematic of which can be seen in figure 1*a*. The system is composed of three components: a sensor system, a data acquisition system (DAS) and a PC with the control and data processing software. The sensor arrangement used in this study consists of four horizontal sensor planes. Each plane is made up of 16 stainless steel electrodes fitted around the circumference of the tank. A conductivity profile is achieved by the DAS injecting an AC current between an adjacent electrode pair, and the resulting voltage is measured between all other electrode pairs. The injection current then shifts to the next electrode pair and this operation continues until a full rotation occurs. A full rotation constitutes 1 frame and is completed approximately once every 6 s. The resultant 104 voltage measurements for each of the four planes are received by the DAS and then transmitted to the PC installed with ITS systems p2+ v. 7.3 software (ITS, Manchester, UK) which uses a non-iterative image reconstruction algorithm (Linear Back Projection) to process the data collected by the DAS. For each plane, the raw voltage measurements are transformed into a two-dimensional conductivity tomogram. The conductivity tomograms represent the cross-sectional distribution of electrical conductivity of the contents of the tank. Each tomogram consists of a 20 × 20 square grid; however, as some pixels are outside the circumference of the tank, the imaging software provides 316 individual conductivity values in each plane, equating to 1264 conductivity values totally over the four planes. Each plane is 4.8 cm apart, taking the floor of the tank to be *H* = 0, the lowest plane (P4) is situated at *H* = 4.1 cm and the highest plane (P1) is situated at *H* = 18.5 cm. This means that the observed mixing chamber, in which the pervasion of the tracer front can be monitored, is a cylindrical volume with a diameter of 20 cm and a height of 14.4 cm.

The experiments involved injecting a 40 ml tracer of 0.3 wt% xanthan gum solution seeded with 1 g of dissolved NaCl. The tracer was injected at the tip of the jet nozzle at time *t* = 0. The evolution of mixing corresponds to the spread of the tracer front and in turn areas of high conductivity in the conductivity profile. The distinction between active mixing region and inactive volume (high conductivity versus low conductivity) is made at a threshold conductivity value. A reference conductivity (*C*_ref_, mS cm^−1^) was set before injecting the tracer. Every subsequent individual conductivity measurement at time *t*, *C_i(t)_* (mS cm^−1^) is normalized with the reference conductivity, and each individual reading is denoted by unitless *σ_i_*, such that

2.1 $$\sigma_{i}=\frac{C_{i(t)}}{C_{\mathrm{ref}}},$$

where *i* =  1 to 1264 for an individual frame. The evolution of mixing corresponds to the spread of the tracer front and in turn areas of high conductivity in the conductivity profile. The distinction between active mixing region and inactive volume (high conductivity versus low conductivity) is made at a threshold conductivity value σ*. The threshold conductivity was calculated as proportional to the conductivity of the bulk fluid when the NaCl tracer is fully dispersed.

2.2 $$\frac{m_{\mathrm{NaCl}}}{V_{T}}=c\propto \sigma^{*},$$

where *m*_NaCl_ is the mass of NaCl (g), *V*_T_ is the total tank volume (m^3^) and *c* is the final concentration of the NaCl in the vessel (g m^−3^). σ* was measured at the end of the experiment after the contents of the tank were mechanically agitated at high shear rates to ensure uniform distribution of NaCl, at time *t* =  t*. Taking this as a final well-mixed frame, it was used with the reference frame to measure all other frames to determine the degree of mixing.

The measurements available from the DAS can be converted to three-dimensional tomograms using Slicer-Dicer software (Pixotec, USA). A representative tomogram is shown in figure 1*b*; where the arrows signify the suction and injection points, the blue region signifies the inactive region, while the red region signifies the well-mixed active region. The software also allows preferential view of the given domain. In figure 1*c*, the tomogram of the tracer-rich domain is shown.

Mixing is monitored in real time by measuring the decay of inactive volume, *V*_i_ (m^3^), as a percentage of total volume in the observed mixing chamber, *V* (m^3^). *V_i_/V* can then be plotted over the dimensionless timescale *N_t_* [16]. The dimensionless timescale is defined as follows:

2.3 $$N_{t}=\frac{t}{t_{H}}=\frac{\dot{Q}t}{V_{T}},$$

where *t* is the process time, *t*_H_ is the hydraulic retention time ($t_{H}=V_{T}\text{/}\dot{Q}$) and $\dot{Q}=0.25\pi d_{j}^{2}v_{j}$ is the recirculation rate. It was deemed that when 90% of the liquid in the vessel is above the threshold conductivity, $t=t_{\theta }$*,* the mixing process was complete. It has been found that ERT experiments are reproducible within 5% error.

Table 2 shows the four nozzle configurations used in this study, for the sake of symmetry, the nozzles were always placed at half liquid height and along the centre plane of the tank. It is important to note that the ↕ denotes a configuration in which the jet and suction nozzle are placed as close as possible to one another. Furthermore, configurations with arrows closest to the wall denote a configuration in which the nozzles are flush with the walls of the tank.

**Table 2.** Basic schematic of nozzle configurations, where ↓ denotes the jet nozzle, ↑ denotes the suction nozzle and ↕ denotes configurations in which jet and suction are as close as possible.

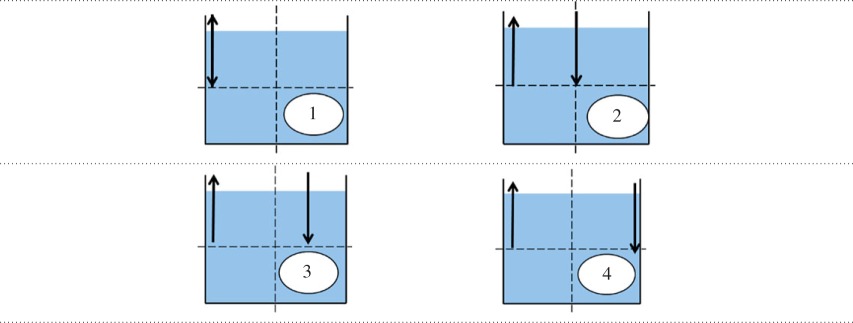

## 3. Results and discussion

**Figure 2.**
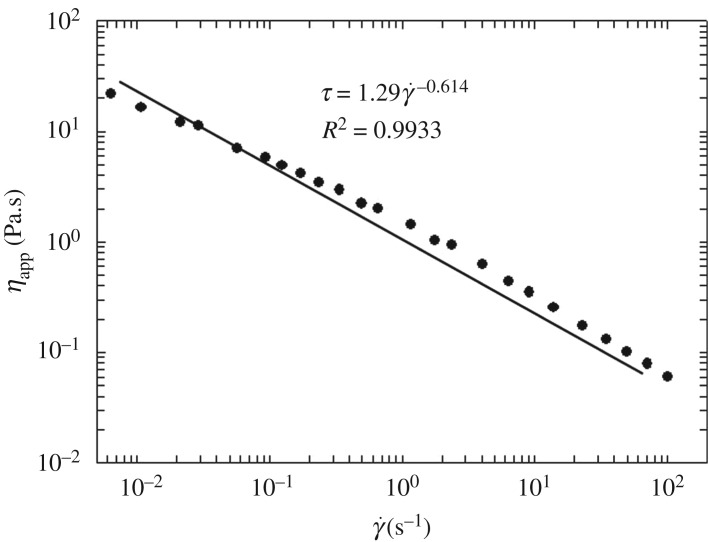
Log scale rheogram of 0.3 wt % XGKT solution with power-law fit.

The rheological response of the liquid used in this study is shown in figure 2, where the markers represent experimental observations. It can be observed from figure 2 that the rheological behaviour of the liquid could be reasonably described by a power-law model where

3.1 $$\eta_{\mathrm{app}}=k{\dot{\gamma }}^{1-n}$$

where *η*_app_ is the non-Newtonian apparent viscosity (Pa.s) and $\dot{\gamma }$ the is shear rate (s^−1^), which are related to each other via the flow consistency index *k* (= 1.29 Pa.s^n^) and the flow behaviour index *n* (= 0.386).

The non-Newtonian Reynolds number at the outlet of the injection nozzle, adapted from Metzner & Reed [17], is calculated as follows:

3.2 $$Re_{j-PL}=\frac{\rho d_{j}^{n}v_{j}^{2-n}}{k{\left((3n+1)\text{/}4n\right)}^{n}8^{n-1}}.$$

The Reynolds number for all of the runs is kept constant at 247, as this is the Reynolds number at the point of injection, it is the maximum Reynolds number reached throughout the system.

**Figure 3.**
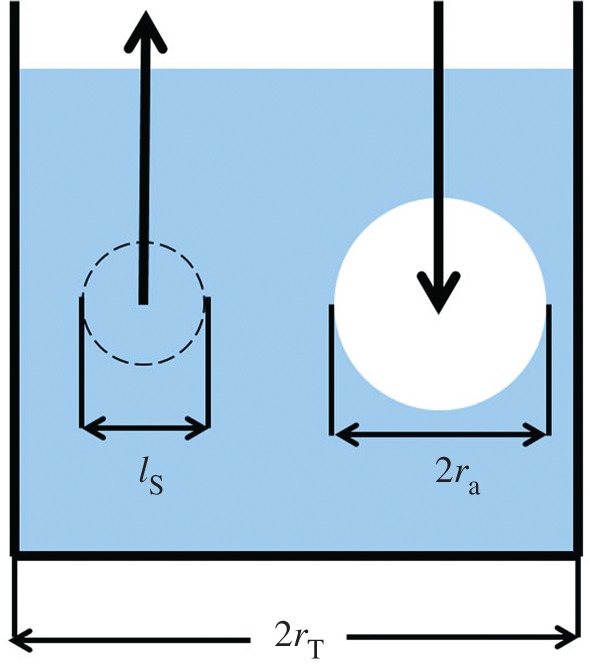
A general diagram representing the quantities of *l*_s,_ *r*_a_ and *r*_T_. (Note: this configuration was not used in this study.)

As mentioned before, we are interested in understanding how the position of the suction nozzle influences the mixing regimes. In order to do so, we estimate the average length-scale (*l*_s_) over which the perturbations due to the suction decay for a given suction velocity *v*_s._ Evidently, as the tracer field spreads with time, the length scale $\left(r_{T}-r_{a}\right)$, where *r*_T_ is the radius of the tank and *r*_a_ is the time-dependent radius of the boundary of the tracer-rich ‘active’ region, approaches zero. These quantities are represented visually in figure 3.

It follows that when $(r_{T}-r_{a})=l_{s}$, the flow field will be affected by dynamics near the suction nozzle. A non-dimensional quantity that naturally arises from the argument above is *Λ* which is as follows:

3.3 $$\Lambda =\frac{r_{T}-r_{a}(t)}{l_{s}}.$$

At the beginning of the experiment, for example in configuration 2 in table 2, however, the numerator in equation (3.3) has a fixed value $\{r_{T}-r_{a}(t=0)\}=\Delta S$ that is given by the separation between the nozzles. We assume that $l_{s}\approx d_{s}$, where *d*_s_ is the diameter of the suction nozzle, in the following, because we are interested in assessing in a quantitative manner the possibility of interaction between the flow domains influenced by both the suction port and the injection nozzle. In this sense, the statement $l_{s}\approx d_{s}$ provides a lower limit to the value of *l*_s_. Indeed, if *d*_s_ is decreased, the value of the velocity at the suction port (*v*_s_) would increase (mass flow rate being constant), and *l*_s_ would increase concomitantly as discussed above. Similarly, when *d*_s_ is increased, *v*_s_ will decrease and consequently *l*_s_ can decrease such that $l_{s}<d_{s}$. In the latter case, the length scale over which the suction port influences the flow domain is given by *d*_s_. In this limit, it is possible to rewrite equation (3.3) as follows:

3.4 $$\Lambda_{*}=\frac{\Delta S}{d_{s}}.$$

The above general form is preferable for design purposes because fixed values are used when calculating Λ_*._ Using the above parameter for characterization, the nozzle arrangements in this work can be quantified in terms of number of nozzle diameters. Here, locations are taken from the centre line of the jet, such that in ↕ configurations *ΔS* =  6 mm. A summary of these values can be seen in table 3. These four basic arrangements were chosen as the apparatus does not offer sufficient resolution to measure the effect of mixing on smaller differences in Λ_*_

**Table 3.** Summary of the geometric parameters found in the mixing configurations.

configuration	Λ_*_
1	1
2	16.2
3	24.5
4	32.3

**Figure 4.**
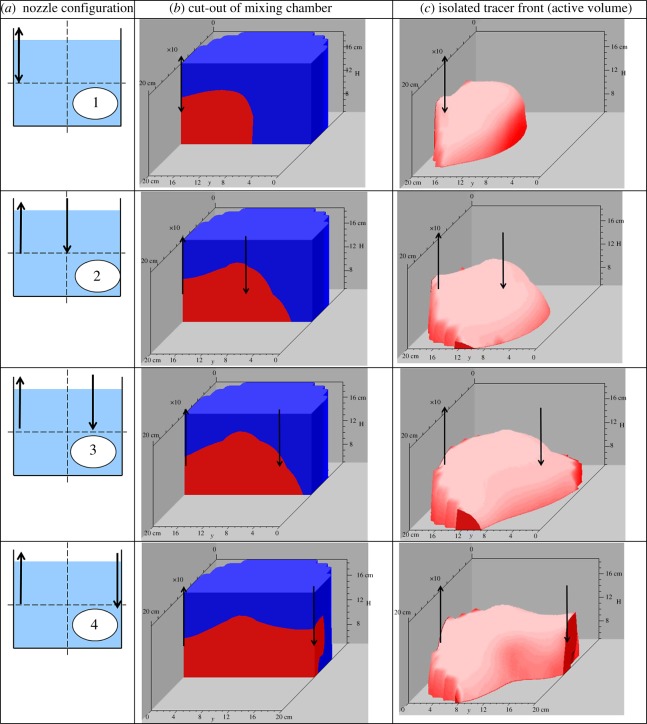
(*a*) Schematic of nozzle configurations used in the study. (*b*) 3D cut-outs of the half of the mixing chamber cut down at the centre plane. (*c*) Isolated images of the active mixing volume in the mixing chamber obtained by Slicer-Dicer software.

Taking the fixed process time of *t* = 2 min, three-dimensional reconstructions of the mixing chamber of 0.3 wt% XGKT solution agitated at *v*_j_ = 0.81 m s^−1^ were created using Slicer-Dicer software (Pixotec, USA) as seen in figure 4.

The middle column shows cut-outs of half the mixing chamber, sliced down the middle plane where the red volume corresponds to the propagation of the tracer front, and thus the active mixing region, and the blue volume corresponds to the inactive volume, where fluid elements are largely stagnant. The flat-face of the half cylinder seen in the middle corresponds to the plane seen in the nozzle configuration column. The H-axis begins at 4.1 cm, because the lowest sensor plane is 4.1 cm above the base of the tank. An inspection of these three-dimensional cut-outs reveals that at *t* = 2 min, the active volume is larger depending on the Λ_*_ value of the nozzle configuration.

The right-hand column shows the isolated tracer front in the full three-dimensional environment of the mixing chamber. Interestingly, it reveals a greater degree of asymmetry than one would expect. It is also interesting to note that the overwhelming majority of the active volume, at this stage, is in between the two nozzles, in the centre plane, but does spread radially outwards. The images seen in figure 4 strongly support the proposal that jet-suction interaction is important in the evolution of active mixing volume. The asymmetry of the active volume can be attributed to the fact that the incoming fluid from the jet nozzle has a preference for the path of least resistance towards the flow field of the suction nozzle. As this phenomenon has not been previously reported in the literature regarding Newtonian fluids, it is thought that this is a non-Newtonian property, given that the viscosity is different at different points of the vessel depending on nozzle location. It is important to note that these are snapshots of evolving active volumes, and are not the final steady states.

**Figure 5.**
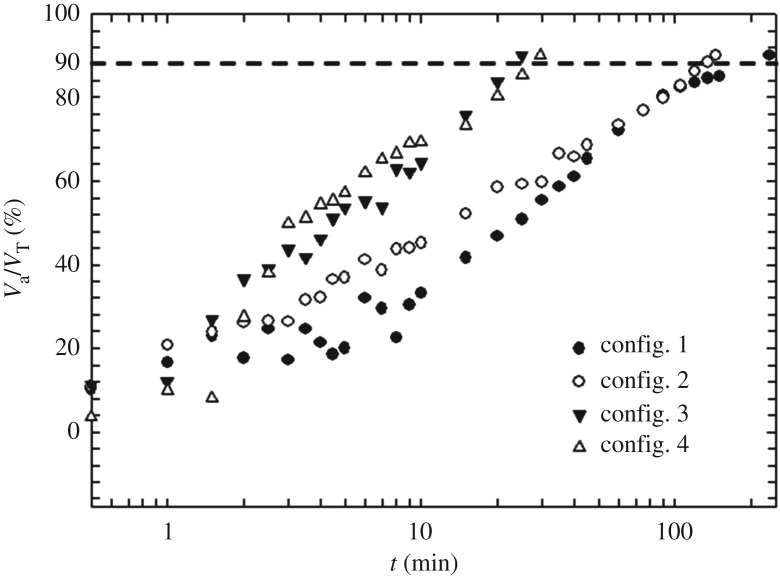
*V*_a_*/V*_T_ versus *t* plot showing the evolution of active volume with time for each configuration.

While it is interesting to investigate the extent of active mixing volume at a fixed time, it is more important for design purposes to investigate the time at which the mixing volume reaches a fixed fraction of the tank. For this study, we have fixed this volume to 90% of *V*_T_. Figure 5 shows a plot of how active volume, *V*_a_ varies with time, until such time it reaches 90% of the fixed volume.

**Figure 6.**
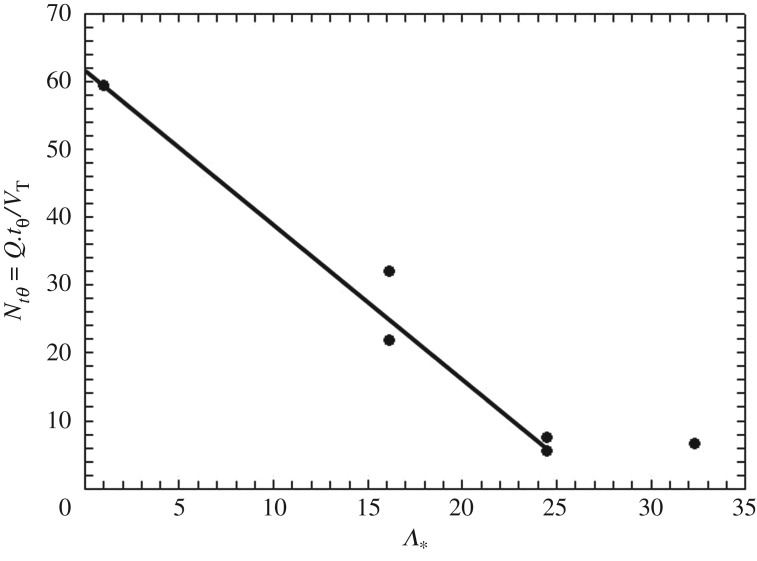
A *N_tθ_* versus Λ_*_ plot showing how the distance between nozzles affects the time at which 0.3 wt% XGKT solution agitated at *v*_j_ = 0.81 m s^−1^ reaches 90% active volume.

Figure 6 shows a plot of $N_{t,\theta }$ which is the mixing time $t_{\theta }\;$ normalized by the hydraulic retention time $t_{H}=V_{T}\text{/}\dot{Q}$, where *V*_T_ is the volume of the tank and $\dot{Q}=0.25\pi d_{j}^{2}v_{j}$ is the recirculation rate. Figure 6 demonstrates how the distance between nozzles affects the time at which 0.3 wt% XGKT solution agitated at *v*_j_ = 0.81 m s^−1^ reaches 90% of *V*_T_. The symbols in the figure are the experimental results. The extra points at Λ* = 16.4 and Λ* = 24.5 are from experiments that are mirror images of the configurations 2 and 3 described in table 2. It can be observed from figure 6 that the jet-suction interplay effect gives rise to a decreasing trend in mixing time as the two nozzles are moved further apart. The experiments have been repeated and based on the data presented in the graph, error has been found within ±5%. Given that the apparatus used only allows for a certain degree of resolution, a lowest order (linear) approximation has been made of this decreasing trend, to which a straight line can be fitted with an *R*^2^ value of 0.9709. Beyond 24.5 nozzle diameters, the plot shows that the distance between the two nozzles has little effect, this is important as it shows that the deleterious effect of flow field distortion ceases to happen when the two nozzles are placed at critical distance.

It follows from figure 6 that for 1 < Λ_* _< 24.5, the lowest order approximation for mixing time can be written as

3.5 $$t_{\theta }=\left(A_{1}+A_{2}\frac{\Delta S}{d_{s}}\right)\left(\frac{D_{T}^{2}H_{T}}{d_{j}^{2}v_{j}}\right),$$

where *A*_1_ and *A_2_* are estimated to be 62.58 and −2.25, respectively, and from linear regression of the experimental data.

The gradual increase of the mixing time as the suction of the injection nozzles is brought closer is interesting because intuition would suggest that as the domains agitated by the nozzle and the suction approach each other, mixing time would decrease. On the contrary, the experiments reported here suggest that the mixing time increases as Δ*S* decreases. We attribute these observations to the ‘short-circuiting’ of the flow field that we have referred to in previous works [15]. In effect, the residence time of injected liquid (momentum) element decreases as Δ*S* decreases and the element escapes the flow domain quickly for small values of *ΔS*. This increases the mixing time. Note that the value of *ΔS* can, in principle, tend to zero, in which case the mixing time would tend to infinity.

Interestingly, the dependence on *v*_j_ appears to be consistent with previous correlation for the mixing time, while those on the *D*_T_ and *H*_T_ are different from the ones shown in equations (1.2) to (1.5). However, unlike the equations described above, equation (3.5) provides a quantification of how the mixing time changes with the relative nozzle location, which was one of the objectives of the current work. The concept of a gradient effect is contrary to Fox & Gex's [2] assertion that the distance between the two nozzles was not important unless the two nozzles were feeding directly into one another. In one sense, Fox and Gex anticipated the phenomenon discussed here without quantifying it. Furthermore, the evidence that this gradient no longer exists when the nozzles are placed at an adequate distance is contrary to Revill's [6] design recommendation that the two nozzles should be placed as far as possible from one another. However, it is concurrent with Hylton & Cummins [9], who also looked at nozzle location and concluded that a liquid jet can fully develop if placed sufficiently far away from the suction nozzle, which leads to better mixing. However, as demonstrated here, the mechanics are richer than those explored in early works and the mixing time can indeed be controlled (to reduce or enhance competition with other transport processes for instance) if needed, by altering the design in a logical way. A jet recirculation mixing system is by its very nature equipped with two energy sources, each with its own zone of influence in agitating the fluid. Competition between the two domains could be used to advantage.

When investigating the impact of nozzle separation on different fluids, the same correlation may hold; however, more experiments would need to be conducted to verify this and this is the subject of further research. The parameters *A*_1_ and *A*_2_ would change as well as the range of Λ_*_ values over which the correlation holds. It follows that *A*_1_ (the intercept) would increase for a more viscous fluid, as it has been shown by the authors elsewhere [18] that a higher viscosity leads to a longer mixing time for a giving mixing configuration. *A*_2_ (the slope) is likely to decrease for a fluid with a higher viscosity, as the separation of nozzles is likely to have a lesser effect for more viscous fluids. The presence of a yield stress would probably shift the range of Λ_*_ over which the correlation holds to the right, as the authors have shown elsewhere [15]; if the nozzles are too close, it leads to cavern formation, thus infinite mixing time, as the vessel will never be fully mixed.

## 4. Conclusion

This study has shown that in vessels using submerged recirculating jets for agitation purposes, the distance between the suction and injection ports can be used to control the mixing time, for the same value of injection velocity and when the liquid properties are kept constant. A non-dimensional quantity is proposed to quantify the degree to which mixing time is enhanced/delayed. The experiments conducted here demonstrate that there is a critical value below which the separation between the suction and injection ports is influential on the mixing time. However, above the critical value, the separation between the ports does not affect the mixing time. The work also suggests an empirical correlation to predict the mixing time under conditions where ‘short-circuiting’ of the flow is dominant, which retains the same dependence of mixing time on the injection velocity and the tank diameter as one of the correlations proposed previously and reviewed here. In this manner, it enhances our capability of designing mixing tanks that use submerged recirculating jets for agitation.

## Data accessibility

The code and datasets supporting this article are available at https://doi.org/10.6084/m9.figshare.4663993.
